# Breastfeeding and the origins of health: Interdisciplinary perspectives and priorities

**DOI:** 10.1111/mcn.13109

**Published:** 2020-11-19

**Authors:** Meghan B. Azad, Nathan C. Nickel, Lars Bode, Meredith Brockway, Amy Brown, Christina Chambers, Camie Goldhammer, Katie Hinde, Michelle McGuire, Daniel Munblit, Aloka L. Patel, Rafael Pérez‐Escamilla, Kathleen M. Rasmussen, Natalie Shenker, Bridget E. Young, Luisa Zuccolo

**Affiliations:** ^1^ Department of Pediatrics and Child Health University of Manitoba Winnipeg Manitoba Canada; ^2^ Developmental Origins of Chronic Diseases in Children Network (DEVOTION) Children's Hospital Research Institute of Manitoba Winnipeg Manitoba Canada; ^3^ Manitoba Interdisciplinary Lactation Centre (MILC) Winnipeg Manitoba Canada; ^4^ Human Capital & Economic Opportunity Global Working Group, Center for the Economics of Human Development University of Chicago Chicago Illinois USA; ^5^ Department of Community Health Sciences and Manitoba Centre for Health Policy University of Manitoba Winnipeg Manitoba Canada; ^6^ Department of Pediatrics and Larsson‐Rosenquist Foundation Mother‐Milk‐Infant Center of Research Excellence University of California San Diego La Jolla California USA; ^7^ Department of Public Health, Policy and Social Sciences and Centre for Lactation, Infant Feeding and Translation Swansea University Swansea UK; ^8^ Mommy's Milk Human Milk Research Biorepository, Center for Better Beginnings University of California San Diego San Diego California USA; ^9^ Indigenous Breastfeeding Counsellor Seattle Washington USA; ^10^ Center of Evolution and Medicine and School of Human Evolution and Social Change Arizona State University Tempe Arizona USA; ^11^ Margaret Ritchie School of Family and Consumer Sciences University of Idaho Moscow Idaho USA; ^12^ Department of Paediatrics and Paediatric Infectious Diseases, Institute of Child's Health Sechenov First Moscow State Medical University Moscow Russia; ^13^ Inflammation, Repair and Development Section, National Heart and Lung Institute Imperial College London London UK; ^14^ inVIVO Planetary Health Worldwide Universities Network (WUN) West New York New Jersey USA; ^15^ Department of Pediatrics, Section of Neonatology Rush University Children's Hospital Chicago Illinois USA; ^16^ Department of Social and Behavioral Sciences, Yale School of Public Health Yale University New Haven Connecticut USA; ^17^ Division of Nutritional Sciences Cornell University Ithaca New York USA; ^18^ Department of Surgery and Cancer Imperial College London London UK; ^19^ Human Milk Foundation Harpenden UK; ^20^ Division of Allergy and Immunology, Department of Pediatrics, School of Medicine and Dentistry University of Rochester Rochester New York USA; ^21^ MRC Integrative Epidemiology Unit and Department of Population Health Sciences University of Bristol Bristol UK

**Keywords:** breastfeeding, breastmilk, conflict of interest, human milk, infant feeding, lactation, research methodology

## Abstract

Breastfeeding and human milk (HM) are critically important to maternal, infant and population health. This paper summarizes the proceedings of a workshop that convened a multidisciplinary panel of researchers to identify key priorities and anticipated breakthroughs in breastfeeding and HM research, discuss perceived barriers and challenges to achieving these breakthroughs and propose a constructive action plan to maximize the impact of future research in this field. Priority research areas identified were as follows: (1) addressing low breastfeeding rates and inequities using mixed methods, community partnerships and implementation science approaches; (2) improving awareness of evidence‐based benefits, challenges and complexities of breastfeeding and HM among health practitioners and the public; (3) identifying differential impacts of alternative modes of HM feeding including expressed/pumped milk, donor milk and shared milk; and (4) developing a mechanistic understanding of the health effects of breastfeeding and the contributors to HM composition and variability. Key barriers and challenges included (1) overcoming methodological limitations of epidemiological breastfeeding research and mechanistic HM research; (2) counteracting ‘breastfeeding denialism’ arising from negative personal breastfeeding experiences; (3) distinguishing and aligning research and advocacy efforts; and (4) managing real and perceived conflicts of interest. To advance research on breastfeeding and HM and maximize the reach and impact of this research, larger investments are needed, interdisciplinary collaboration is essential, and the scientific community must engage families and other stakeholders in research planning and knowledge translation.

**Abbreviations**
HM: human milk
DHM: donor human milk

**Key messages**

- Breastfeeding is critically important to maternal, infant and population health, yet we still lack a fundamental understanding of human milk (HM) composition and most mother–infant dyads do not achieve breastfeeding recommendations.
- This field of transdisciplinary research is challenged by methodological limitations and the need to inform, yet remain distinct from, breastfeeding advocacy.
- To advance research related to breastfeeding and HM science and maximize its reach and impact, the scientific community must engage families and other stakeholders in research planning and knowledge translation and properly manage COI. Larger investments are needed, and interdisciplinary collaboration is essential.

## 1 WORKSHOP RATIONALE AND METHODS

Breastfeeding provides a constellation of health benefits for mothers and infants. Considering the abundance of evidence and long‐standing global recommendations to support breastfeeding, it is surprising that we still do not understand the underlying biological mechanisms of these benefits, and it is concerning that most mother–infant dyads do not achieve breastfeeding recommendations. To address these issues, we convened a workshop of experts in the field of breastfeeding and human milk (HM). The workshop focused on two main areas of concern emphasized by participants through priority‐setting exercises before and during the workshop: the need for more interdisciplinary research in this field and the need to address counterproductive tensions between breastfeeding research and advocacy efforts.

### 1.1 Interdisciplinary research

Knowledge about the health effects of breastfeeding and HM has typically come from disparate lines of research in the basic, clinical and social sciences. Basic scientists have advanced our understanding of milk composition through laboratory research (e.g., Andreas, Kampmann, & Mehring Le‐Doare, 2015; Boix‐Amorós et al., 2019; Doherty et al., 2018; Fitzstevens et al., 2017; Gay et al., 2018; Waidyatillake et al., 2018), whereas social scientists and clinical researchers have studied the complex social, clinical, economic and institutional factors that influence breastfeeding at the individual and population levels (e.g., Nickel et al., 2014; Pérez‐Escamilla, Martinez, & Segura‐Pérez, 2016; Schindler‐Ruwisch et al., 2019; Temple Newhook et al., 2017). For the most part, these advances have occurred with minimal interaction between disciplines, limiting the translation and impact of this research. It is encouraging to see that interdisciplinary research in this field is increasing thanks to recent efforts by international organizations (e.g., International Society for Research in Human Milk and Lactation and Academy of Breastfeeding Medicine), research centres (e.g., Carolina Global Breastfeeding Institute, Manitoba Interdisciplinary Lactation Centre [MILC] and Mother‐Milk‐Infant Centre of Research Excellence [MOMI‐CORE]) and initiatives (e.g., Lactation, Infant Feeding and Translational Research [LIFT], International Milk Composition Consortium [IMiC] and Breastmilk Ecology‐Genesis of Infant Nutrition: Understanding Human Milk as a Biological System [BEGIN])—however, there is still much room for improvement and expansion of the interdisciplinary efforts in HM and lactation research.

### 1.2 Counterproductive tensions

Like other areas of study, the major sources of funding for research on HM and infant feeding originate from governments, philanthropic or charitable foundations and other non‐profit organizations, and industry. Many HM and breastfeeding researchers carefully manage potential conflicts of interest (COIs) with industry. Others choose to avoid financial COI altogether, and some also recognize and uphold the World Health Organization (WHO) Code of Marketing of Breastmilk Substitutes, which, although relevant to breastfeeding, is focused on the marketing of commercial products and not research governance. Scientists on both ends of this spectrum have been publicly shamed for their decisions. Aside from directly impacting the targeted individuals and areas of investigation, these dynamics may discourage young scientists from entering the field. Breastfeeding and HM researchers must also navigate increasingly complex social challenges when translating their research because social media is increasingly used to perpetuate misinformation or biased interpretations of scientific evidence about breastfeeding and infant formula. Correcting misinformation is challenging and time consuming and can detract from research activities. Although these challenges are not entirely unique to breastfeeding and HM research, they are heightened in this field due to the emotion associated with infant feeding decisions.

### 1.3 Methods

To discuss these challenges facing breastfeeding and HM researchers, a multinational group of breastfeeding and HM researchers from diverse disciplines and career stages gathered in February 2019 in Winnipeg, Canada, for a workshop titled ‘Breastfeeding and the Origins of Health: Interdisciplinary Perspectives and Priorities’. The mandate of this workshop was to identify and discuss research priorities and anticipated breakthroughs in breastfeeding or HM research (Section 2); discuss the perceived barriers and challenges to achieving these breakthroughs (Section 3); and outline a plan of action towards supporting and maximizing the impact of future breastfeeding and HM research (Section 4). Participants were invited on the basis of their expertise in breastfeeding and HM research or practice, with consideration for equity, diversity and inclusion across disciplines, settings and career stages. Not everyone who was invited was able to attend, and a few declined participation precisely because of the tensions the workshop aimed to address. Participants completed a preworkshop survey to guide preparations. The workshop consisted of short presentations, interactive priority‐setting exercises, group discussions and breakout sessions. Local stakeholders (researchers, trainees and healthcare practitioners) attended some sessions and contributed to discussions. During the workshop, participants codeveloped the outline of this paper and formed writing groups to draft each section in response to the workshop's stated aims. Writing continued after the workshop through a collaborative and iterative process involving all invited participants as co‐authors.

## 2 PRIORITIES AND ANTICIPATED BREAKTHROUGHS IN BREASTFEEDING AND HUMAN MILK RESEARCH

### 2.1 Using implementation science to address low breastfeeding rates and breastfeeding inequities

#### 2.1.1 Low breastfeeding rates, inequities and barriers

Breastfeeding is among the most cost‐effective public health interventions available, providing protection against several short‐ and long‐term health conditions for both mother and infant (Victora et al., 2016), which reduces healthcare costs (Rollins et al., 2016). The WHO recommends that all infants be exclusively breastfed for around 6 months and continue breastfeeding with complementary foods until 2 years or beyond (WHO, 2003), yet by 6 months of age, only 58% of US infants are breastfed and just 25% are exclusively breastfed (Centers for Disease Control and Prevention, 2018). Rates are lower in the United Kingdom (34% any breastfeeding at 6 months), the Netherlands (32%) and France (23%) (Victora et al., 2016). In many settings, breastfeeding rates are even lower among infants born to minority and/or low‐income mothers, which may contribute to long‐term health inequities in these marginalized populations (Anstey, Chen, Elam‐Evans, & Perrine, 2017; Merewood et al., 2019; Patel et al., 2019).

Barriers to breastfeeding include stigma, lack of support and structural factors that disproportionately affect marginalized populations (e.g., lack of breastfeeding education and support services and inadequate maternity leave policies) (Nickel et al., 2014). Social determinants of health and cultural factors also influence breastfeeding outcomes (Byrd, Balcazar, & Hummer, 2001; Cattaneo, 2011; Celi, Rich‐Edwards, Richardson, Kleinman, & Gillman, 2005; Dubois & Girard, 2003; Patel et al., 2019). Of great concern, the breastfeeding gap within populations is widening (Li et al., 2019; Logan et al., 2016; Nickel et al., 2014). It is critical to understand the reasons for this disparity and to collaboratively develop context‐specific strategies to address them.

#### 2.1.2 Implementation science

Addressing low breastfeeding rates and breastfeeding inequities requires *implementation science* (Pérez‐Escamilla & Hall Moran, 2016) to translate research into evidence‐based advocacy efforts, policies and large‐scale programmes. Implementation science involves mixed‐methods approaches to design, evaluate and scale up effective programme innovations, and strategies to enhance the use of existing knowledge, tools and frameworks based on a systems thinking approach (Tumilowicz et al., 2019). Coordinated efforts by multidisciplinary teams are required to execute planning, collaboration, monitoring and adjustments. Implementation science has been applied successfully to scale up effective breastfeeding programmes across world regions using the breastfeeding gear model (Pérez‐Escamilla, Curry, Minhas, Taylor, & Bradley, 2012) and building upon evidence‐based interventions (Merewood et al., 2019; Nickel, Taylor, Labbok, Weiner, & Williamson, 2013; Pérez‐Escamilla et al., 2016).

### 2.2 Improving awareness of evidence‐based benefits, challenges and complexities of breastfeeding among health practitioners and the public using effective messaging platforms

#### 2.2.1 Lack of awareness and competing/inconsistent messaging

Evidence‐based and culturally competent engagement about breastfeeding remains a constant challenge, particularly when contrasted by the sophisticated messaging strategies used by infant formula companies (Seals Allers, 2018). This challenge is compounded by a lack of formal education about lactation and breastfeeding support for most healthcare professionals (Freed et al., 1995; Younger Meek, 2019). There is also a lack of rigorous science investigating the implications of breastfeeding and/or HM on infant health. At the same time, health‐focused research and messaging often fail to acknowledge that many women want to breastfeed for cultural or religious reasons, or simply because it is a physiological norm and a reproductive right, regardless of any health benefits (Brown, 2018). This constellation of challenges has resulted in public confusion and inconsistent messaging regarding breastfeeding and HM.

#### 2.2.2 Reaching everyone with appropriate messaging

Supporting breastfeeding is a societal responsibility (Rollins et al., 2016). Mothers and infants are underserved by societies that deprive families of the autonomy and information to make evidence‐based decisions about infant feeding, invalidate mothers' emotions and desires to breastfeed (or not), default to infant formula rather than effectively supporting breastfeeding and undervalue the time and energy that women dedicate to breastfeeding (Brown, 2018). Messages should not focus on the individual mother alone; they should be adapted for traction across all stakeholders that influence breastfeeding success—from grandparents and clinicians to employers, business owners and political bodies. It is also important to ‘normalize’ breastfeeding for the next generation of families through embedding breastfeeding education in school curriculums (Glaser, Roberts, Grosskopf, & Basch, 2015).

Messages must be culturally sensitive and recognize that, in some countries, inequities in breastfeeding have resulted from historical trauma and discrimination against marginalized communities (Asiodu & Flaskerud, 2011; Heart, Chase, Elkins, & Altschul, 2011). Effective initiatives built within these communities are foundational models for achieving inclusive care (e.g., Momma's Village, Indigenous Breastfeeding Counsellor, and Reaching Our Sisters Everywhere: African American Breastfeeding Blueprint) (Bugg & Bugg, 2013). Healthcare providers (Pound, Moreau, Hart, Ward, & Plint, 2015) and policymakers must be properly and comprehensively trained, as messaging to promote breastfeeding will have limited success without equitable policies that support and protect breastfeeding at the individual, institutional and societal levels.

#### 2.2.3 Leveraging social media and online communities

Social media platforms provide a global medium to amplify public health campaigns, influence health behaviours and establish social norm (Giustini, Ali, Fraser, & Kamel Boulos, 2018; Merchant, 2020). Social media can be used to share educational and supportive messaging about breastfeeding and HM (Marcon, Bieber, & Azad, 2018; Price et al., 2018); however, it can also facilitate dissemination of pseudoscience and provide a platform for divisive agents (Giustini et al., 2018). Opportunities exist to spread breastfeeding messaging more broadly and effectively using social media (Brown, 2016), smartphone apps (Coughlin, 2016), animations (e.g., bit.ly/2euMoxh), interactive infographics (e.g., human‐milk.com) and popular science writing. Academics studying breastfeeding and HM should make better use of these ‘nontraditional’ forms of knowledge translation or actively engage with messaging experts to maximize the reach and impact of their research.

### 2.3 Studying and supporting alternative modes of human milk feeding: Expressed milk, donor milk and shared milk

#### 2.3.1 Expressed milk

Exacerbated by the lack of a national paid parental leave policy, over 85% of US mothers express (pump) their milk at some point during lactation (Labiner‐Wolfe, Fein, Shealy, & Wang, 2008), including some who solely feed expressed HM (Keim, Boone, Oza‐Frank, & Geraghty, 2017). This practice has also increased in other industrialized nations—for example, in Hong Kong, exclusive pumping increased from 5–8% in 2006 to 18–20% in 2011 (Bai, Fong, Lok, Wong, & Tarrant, 2017).

Feeding bottled HM may not be biologically equivalent to feeding at the breast. Differences have been observed for infant weight gain (Azad et al., 2018), satiety (Li, Fein, & Grummer‐Strawn, 2010), asthma (Klopp et al., 2017) and memory (Pang et al., 2019), suggesting a potential negative impact from the process of bottle feeding and/or reduced bioactivity of expressed HM. However, feeding expressed HM still provides benefits compared with infant formula (Azad et al., 2018; Klopp et al., 2017) and should be encouraged when nursing is not possible or preferred. Future research should capture the complexity of modern HM feeding practices, even among exclusively breast (milk)‐fed infants. As new evidence emerges, guidelines (Eglash & Simon, 2017) may require revision to provide up‐to‐date advice for storing and feeding expressed HM. It is also critical to address the structural barriers that force women to choose between pumping and stopping breastfeeding altogether.

#### 2.3.2 Donor milk and milk sharing

The availability and use of donor HM (DHM) is increasing. In preterm infants, access to DHM (as compared with infant formula) lowers the risk of developing necrotizing enterocolitis (Quigley, Embleton, & McGuire, 2019) and can support the establishment of the mother's own milk supply (Kantorowska et al., 2016; Wilson et al., 2018) but may result in lower growth rates (Quigley et al., 2019). Research is needed to identify best practices, including whether and how pooling (Young et al., 2018) and pasteurizing (Ewaschuk, Unger, Harvey, O'Connor, & Field, 2011) should be conducted to preserve the bioactive integrity. ‘Personalizing’ DHM is another area requiring innovation—for example, by matching DHM on maternal and/or infant characteristics or using mother's own milk to seed the microbiota of DHM (Cacho et al., 2017). Research is also needed to inform prioritization of DHM allocation and improve milk banking processes (Matthews et al., 2019).

The limited access to DHM in most countries has led to a large increase in unregulated informal HM sharing (Palmquist et al., 2019). To prevent potential harms from these practices, a pragmatic approach has been proposed by the Academy of Breastfeeding Medicine (Sriraman, Evans, Lawrence, & Noble, 2018), outlining risks versus benefits to help parents make evidence‐based decisions. Research is underway to address the paucity of evidence available regarding the use of DHM in term infants.

Overall, modern caregivers are actively seeking practical advice (Lupton, 2016) to inform their diverse feeding regimens, and much more research is necessary to provide evidence‐based recommendations. This will require researchers to explore and document alternative feeding modes and engage with diverse stakeholders including breast pump manufacturers, donor milk banks and regulatory agencies.

### 2.4 Using innovative approaches to understand mechanisms of the health effects of breastfeeding and the variability of human milk composition

#### 2.4.1 Human Milk: A complex, dynamic, living tissue

Evidence linking breastfeeding to health benefits for mothers and infants varies across studies, settings and populations—possibly because of methodological differences or variation in HM composition. Milk contains vitamins, minerals, lipids, proteins, carbohydrates, enzymes, hormones, cytokines, antibodies and microRNAs. Milk is also a ‘living tissue’ containing viable human and microbial cells, although their role in infant health is unclear (Witkowska‐Zimny & Kaminska‐El‐Hassan, 2017). The concept of ‘lactotypes’ has been proposed, suggesting that women can be characterized according to their milk composition profile (Munblit et al., 2017) and that variation in *combinations* of milk components rather than *single factors* may be linked with infant health.

#### 2.4.2 Determinants of human milk composition

**FIGURE 1:**
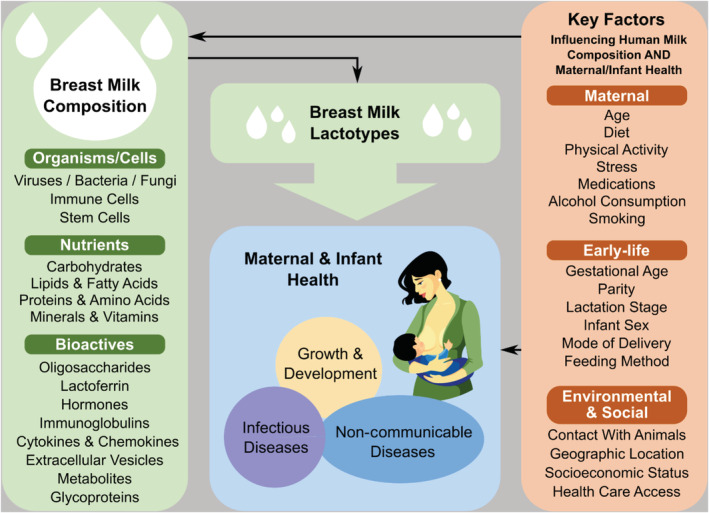
Determinants and consequences of human milk composition

Many HM constituents vary greatly between and within populations, and even within the same individual over time, depending on multiple fixed and modifiable factors (Boix‐Amorós et al., 2019; Bravi et al., 2016)—including maternal age, diet, parity, stage of lactation, metabolic and immune health, physical activity, medications, mode of delivery, length of gestation, infant sex and social networks (e.g., Bravi et al., 2016; Cacho et al., 2017; Meehan et al., 2018; Munblit et al., 2017; Witkowska‐Zimny & Kaminska‐El‐Hassan, 2017) (Figure 1). Genetics are also relevant; for example, single‐nucleotide polymorphisms in the fucosyltransferase and fatty acid desaturase gene clusters are associated with 2′‐fucosyllactose and ω6‐polyunsaturated fatty acid concentrations, respectively (Glaser, Lattka, Rzehak, Steer, & Koletzko, 2011; Meldrum et al., 2018). Genome‐wide association studies have been used for decades in the dairy industry (Fang et al., 2017) and are warranted to examine HM composition.

Box 1 **Sources of heterogeneity in epidemiological studies of breastfeeding**

A) Bias‐inducing limitations (measuring the same effects, but with bias)

- ***Confounding***—The main barrier to inferring causality in observational studies of breastfeeding is confounding by socioeconomic factors and variables following a socioeconomic gradient (e.g. maternal health and lifestyle). This is because establishing or continuing to breastfeed is strongly associated with socioeconomic status (particularly in high‐income countries), as are many of the health outcomes.
- ***Selection bias***—Studies focused on determinants of breastfeeding duration can be biased if they exclude mothers who do not initiate breastfeeding, particularly if these same determinants also affect initiation (Paternoster, Tilling, & Davey Smith, 2017).
- ***Publication bias***—Compared to studies suggesting no relationship between breastfeeding and a health outcome, those showing positive associations are more likely to be published (Horta & Victora, 2013), thus affecting conclusions drawn in systematic reviews and meta‐analyses.

B) Non‐bias inducing limitations (measuring different effects)

- ***Misclassification of breastfeeding exposures***—Standardized definitions have been proposed for breastfeeding research, but many studies do not apply them (Miliku & Azad, 2018). Ideally, studies should capture and distinguish the following:
  - Duration and exclusivity of breastfeeding
  - Nursing at the breast vs expressed HM (relative proportion of each; storage of expressed milk)
  - Perinatal feeding exposures in hospital
  - Introduction of complementary foods (both age and type/quality of food)
  - If partially breastfed: relative proportion of HM vs infant formula
  - If bottle fed (whether infant formula or HM): feeding style
  - If formula fed: variation in type of infant formula used (e.g. high/low protein, protein source and size, percentage carbohydrate from lactose, addition of pre/probiotics, lactoferrin, milk fat globule membrane, etc.)
- ***Failure to address effect modifiers and interactions***—There may be genuine differences in breastfeeding effects when breastfeeding interacts with setting‐specific cultural/environmental factors. Such interactions are rarely addressed but should be considered. Possible modifiers include the following (though it should be noted that some of these factors could also be confounders).
  - Maternal diet, lifestyle and drug use (prescription or recreational)
  - Maternal physical and mental health
  - Maternal/parental attachment and parenting style
  - Environmental exposures that are mitigated or exacerbated by breastfeeding (e.g. pollution, smoking)
  - Differences in HM composition (see Section 2.3)

Geographic variation in HM composition has also been described (Gay et al., 2018; Kumar et al., 2016; McGuire, Meehan, Brooker, et al., 2017; Munblit et al., 2016; Ruiz et al., 2017) and might reflect optimization of milk for particular environments (McGuire, Meehan, McGuire, et al., 2017). This ‘eco‐homeorhesis’ phenomenon suggests that there is no one‐size‐fits‐all construct for milk composition and could inform strategies to ‘personalize’ HM for particular settings and contexts. However, these differences in milk composition may reflect historical exposures—such as pathogens that are no longer common to the region. Thus, milk composition likely reflects the sum of previous and current circumstances. Investigation of this important concept requires tightly controlled studies with standardized collection of milk and health data on a global scale.

#### 2.4.3 Relating variation in human milk composition to infant health

Current evidence relating HM components to infant health is limited by several factors related to study design and methodology (see Section 3.1) and frequently focuses on single HM constituents. Future studies should investigate a wider selection of components and engage experts in statistics and data science to consider the interactions between them. Translational approaches are needed to build on these observations with mechanistic studies. This will require randomized controlled trials (e.g., milk components as supplements), in vitro experiments and animal models. Systematic reviews are also needed (Doherty et al., 2018; Fitzstevens et al., 2017; Gao et al., 2019; Khaleva et al., 2019; Waidyatillake et al., 2018) to inform future research.

## 3 BARRIERS AND CHALLENGES TO BREASTFEEDING AND HUMAN MILK RESEARCH

### 3.1 Methodological limitations of epidemiological and mechanistic research

Evidence supporting or refuting ‘health claims’ associated with breastfeeding is often conflicting (Doherty et al., 2018; Evenhouse & Reilly, 2005; Munblit et al., 2017; Torregrosa Paredes et al., 2014; Waidyatillake et al., 2018). This is problematic because when claims are publicly refuted, trust in the scientists and health professionals producing and conveying these claims could be eroded, potentially leading to a backlash against researchers and breastfeeding promotion efforts. Robust evidence quantifying specific health effects (or lack thereof) and their mechanisms will be key to producing reliable cost–benefit analyses and advocating for more investment in services to protect, promote and support breastfeeding.

#### 3.1.1 Epidemiological studies of breastfeeding

It is not ethical to randomize breastfeeding, so almost all evidence supporting or refuting breastfeeding or HM feeding comes from observational studies or animal models. Epidemiological studies vary in design, size and setting (e.g., low‐ vs. high‐income countries), and their collective results reflect considerable heterogeneity for many of the outcomes studied (Victora et al., 2016). Heterogeneous results do not necessarily signal a ‘reproducibility crisis’ as there may be genuine differences when breastfeeding interacts with setting‐specific cultural and environmental factors. For example, breastfeeding appears to lessen the negative effect of air pollution and tobacco smoke on development of asthma (Moshammer & Hutter, 2019); thus, the effect of breastfeeding on asthma may appear greater in settings with high rates of these exposures. However, differences in effect estimates could also result from different degrees of bias, which contributes to a lack of reproducibility. Box 1 lists the main sources of heterogeneity in epidemiological studies of breastfeeding. The cluster‐randomized Promotion of Breastfeeding Intervention Trial (PROBIT) (Kramer et al., 2001) offered a rare opportunity to evaluate the causal impact of this programme in Belarus; however, this trial excluded nonbreastfed infants and the results may not be generalizable to other settings and modern feeding regimens (Martens, 2012).

#### 3.1.2 Mechanistic studies of human milk components

HM contains a plethora of nutritional and bioactive components (Section 2.4). Methods may differ between studies, occasionally without appropriate validation for measurement in HM, which is a unique and complex matrix compared with other body fluids and even other types of milk. HM components are often quantified in terms of concentrations as opposed to the cumulative ‘dose’ received by the nursing infant. The dose is more relevant but also more challenging to measure because it requires knowing the volume of milk consumed by the infant. Methods used to obtain and store HM samples are often not reported (factors to consider are shown in Box 2).

### 3.2 Negative personal experiences with breastfeeding can fuel ‘breastfeeding denialism’ and impede research progress and translation

Public discussions about infant feeding in mainstream and social media highlight the deeply personal nature of infant feeding experiences. Women express the joy they experience while breastfeeding and share their struggles and emotional turmoil when they are unable to meet their own breastfeeding goals (Brown, 2018). Negative or ‘denialist’ attitudes towards breastfeeding are sometimes fuelled by individuals with negative personal experiences (Palmer, 2019), which often originate from disempowering interactions with healthcare systems (Brown, 2018). Researchers face complex challenges when discussing the health benefits of breastfeeding because, although advancing research on this topic will ultimately improve health for *all* mothers and infants, it also perpetuates a dialogue that can cause guilt among women who did not breastfeed. These personal biases can impede research progress and impact by influencing the peer review process and the translation of research results. One way to address this challenge is to avoid focusing entirely on the mother–infant dyad and their (in)ability to breastfeed, which ignores the myriad underlying social and structural determinants that affect this process, as discussed in Section 2.2. Another way to address this challenge is to undertake qualitative research focused on understanding the lived experiences of families who have struggled with breastfeeding (Spencer, 2008). It is also important that women unable to breastfeed are supported through research on alternative feeding methods and responsive bottle feeding.

### 3.3 Distinguishing and aligning advocacy and research efforts

**FIGURE 2:**
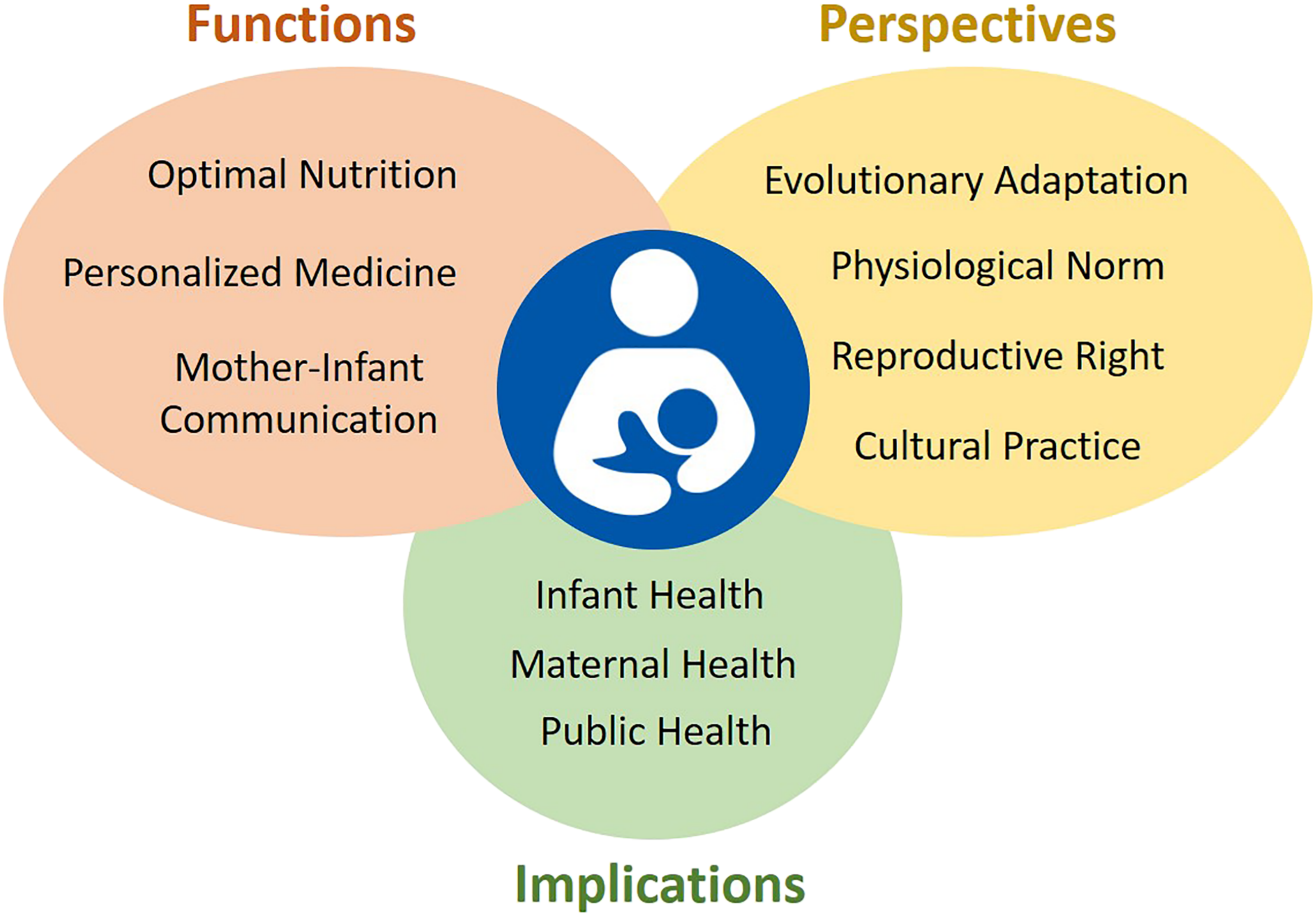
The biopsychosocial construct of breastfeeding: functions, perspectives and implications

Advocacy is central to advancing public health agendas, including breastfeeding (Michaud‐Létourneau, Gayard, & Pelletier, 2019; Pérez‐Escamilla et al., 2012; Rosen, 1993). In parallel, marketing messaging by industry has been used heavily to advocate for infant formula (Robinson, Buccini, Curry, & Pérez‐Escamilla, 2018). Although it is generally agreed that advocacy should be based on research, it can be difficult to reach consensus on the sufficient level of evidence. In the case of breastfeeding and HM, there is often a lack of consensus stemming from the limitations and inconsistency of current evidence (Section 3.1), the complex nature of HM composition (Section 2.4) and the personal biases of individual experts. Moreover, breastfeeding itself is a complex construct (Figure 2) that can be considered as a health issue for both mother and infant, a basic human right for the infant and a reproductive right for the mother. Breastfeeding can be approached from clinical, public health or anthropological perspectives and can be viewed as nutrition, ‘personalized medicine’ or a means of maternal–infant nurturing interactions. Given these complexities and nuances, it is not surprising that experts can struggle to reach consensus on the type of evidence needed for informing advocacy efforts.

Box 2 **Selected sources of heterogeneity in human milk research**

- Full feed expression vs foremilk only (beginning of feed) vs. hindmilk only (end of feed) vs. mid‐feed
- Time of day
- Method of collection (hand pump, electric pump, or hand expressed) and container (material, sterilized or not)
- Method used to clean (or not) the breast and/or pump
- Collection and storage containers (glass, plastic, clear, opaque, amber)
- Temperature and time in storage
- Maternal characteristics (feed‐specific), prandial state/diet/medications/drug use
- Stage of lactation (age of infant)
- Samples from a single feed or pooled samples from multiple feeds
- Thawing, processing and mixing protocol
- Validity of assay in human milk
- Capture milk volume to calculate dose consumed

#### 3.3.1 Unsubstantiated or poorly substantiated claims

Infant formula manufacturers have been known to make unsubstantiated claims for marketing purposes (Munblit, Crawley, Hyde, & Boyle, 2020). For example, some products fortified with DHA are advertised as improving cognitive development, yet there is little rigorous evidence to support this claim (Moon, Rao, Schulzke, Patole, & Simmer, 2016). The addition of biologically active ingredients to infant formulas without clear evidence of long‐term safety or benefit is an issue of increasing concern (Abrams & Daniels, 2019; Kaneko, Fasano, & Choudhuri, 2019).

Claims may also be unsubstantiated in breastfeeding advocacy and promotion efforts. There is a danger that interesting new clinical or laboratory findings related to HM (e.g., the presence of stem cells) may be used prematurely by advocacy groups before the direct benefits to infant or maternal health are understood. Such claims can inadvertently undermine the support of breastfeeding by implying that further research is not needed and giving the impression that research in this field is not sufficiently rigorous. Such claims may later be used by industry to justify adding new ingredients to infant formula without appropriate evidence.

#### 3.3.2 Supporting advocacy with evidence

As a general strategy, population‐wide efforts to improve science literacy will help individuals understand research in context and appreciate that all research has limitations. Targeted efforts are being made to ensure that breastfeeding and HM‐related advocacy is based on rigorous evidence. For example, great improvements have occurred in the process for developing evidence‐informed infant feeding guidelines through the WHO (2012). The United States Department of Agriculture (USDA) has developed the Nutrition Evidence Systematic Review (formerly known as the Nutrition Evidence Library) to help systematize the grading of dietary recommendations, including for infants (see https://www.fns.usda.gov/resource/nutrition-evidence-systematic-review). The Cochrane Collaboration launched a special collection of systematic reviews on support and care for breastfeeding women, treatment of breastfeeding‐associated problems and breastfeeding infants with additional needs (see https://www.cochranelibrary.com/collections/doi/10.1002/14651858.SC000027/full).

Advocacy efforts should be grounded in evidence, and research efforts should involve knowledge users to inform advocacy. Unjustified claims often result from genuine misunderstanding or poor knowledge translation. Researchers can support advocacy efforts by providing clear evidence summaries and speaking up when findings are inappropriately used for advocacy. In addition, researchers can contribute by evaluating advocacy strategies to objectively determine their efficacy (Brindis & Gardner, 2017; Glass, 2017) and actively participating in initiatives that lead to effective public policy and advocacy recommendations (Pérez‐Escamilla et al., 2012).

### 3.4 COIs in breastfeeding and human milk research

COIs in research put the process at risk by potentially biasing a researcher's professional judgement (Suter & Cormier, 2015). COI can emerge across a variety of dimensions when an individual has a personal, professional or financial interest that could affect how they carry out or interpret their work. Here we focus on financial COI and offer a discussion of the challenges and opportunities afforded by working with industry partners for breastfeeding and HM research. Workshop discussions highlighted the diversity of opinions on this topic.

#### 3.4.1 Industry sponsorship of breastfeeding and human milk research can lead to bias and incorrect public health messaging

Over the past century, many industries have funded research as a strategy for gaining public credibility and acquiring market share (Bekelman, Li, & Gross, 2003; Flacco et al., 2015; Lexchin, Bero, Djulbegovic, & Clark, 2003; Lundh, Lexchin, Mintzes, Schroll, & Bero, 2018). This practice extends to breastfeeding and HM science, where the infant feeding industry invests heavily in breastfeeding and HM research (Shenker, 2018; Van Tulleken, 2018). Industry funding may influence decision making in academic healthcare settings on an unconscious level, reflecting ‘motivational bias’ (Dana & Loewenstein, 2003). The act of declaring COI may actually exaggerate rather than mitigate this form of bias (Cain, Loewenstein, & Moore, 2005, 2010). Scientists who ignore the risks of motivational bias can inadvertently facilitate the dissemination of incorrect public health messages (Bekelman et al., 2003; Campbell, Louis, & Blumenthal, 1998; Smith, 2006; Thompson, 1993). There is no guarantee that open declarations of COI will prevent such bias when researchers accept grant funding (whether or not it is restricted) from companies with vested interests in the outcomes generated. Further, a randomized study (Sharek, Schoen, & Loewenstein, 2012) showed that the impact of motivational bias may extend to the development and evaluation of COI policies when these policies are developed by those closest to the field. Involving impartial organizations and ethicists in the development of COI policies could help prevent this potential bias.

#### 3.4.2 Industry partnerships can contribute meaningfully to breastfeeding and human milk research and produce unbiased results when COIs are effectively managed

Industry partnerships can be important for the advancement of science and translation of discoveries. Throughout this process, however, it is important to manage COI to ensure that they do not bias study findings or the dissemination of results. It is critical that researchers fully disclose their funding sources and the nature of any potential COIs and apply appropriate study designs and oversight to ensure the validity and integrity of their study's results. This includes standardization of methods, use of appropriate control groups, and application of statistical techniques and models to account for confounders. In clinical trials, randomization and blinding are standard methods applied to prevent COI from influencing results. Most academic institutions do not allow funders (e.g., granting agencies, foundations or corporate entities) to influence whether or not research findings can be published and have policies in place to prevent funders from influencing study findings and publication. Moreover, most scientists publish their studies in refereed journals, adding important, though imperfect (Dyer, 2019; John, Loewenstein, Marder, & Callaham, 2019), layers of protection against COI.

#### 3.4.3 Moving forward

Responses to concerns surrounding real and perceived COI need to be balanced. Given that public research funding is limited, particularly for maternal and infant health research (Johnson, 2019), some scientists are concerned that strictly refusing all interactions with industry could hinder research progress and limit the ability of researchers to hold scientific meetings, unless alternative forms of funding are made available. Other scientists perceive that ignoring or mismanaging COI concerns could jeopardize research integrity in this field. Ultimately, the enduring solution to potential problems related to COI will involve a combination of avoiding (where possible and prudent) or acknowledging, declaring and rigorously managing COI.

## 4 ROADMAP AND CONCLUSIONS

### 4.1 How to achieve breakthroughs and overcome barriers

There is a growing recognition of the importance and exquisite complexity of HM as a living tissue promotes infant health. At the same time, there is an increasing appreciation that breastfeeding practices and HM composition are influenced by psychosocial factors and the social and structural determinants of health. These findings indicate a need for scientists to adopt a holistic view of breastfeeding and HM and establish interdisciplinary collaborations to carry out this research. In particular:

- To address breastfeeding inequities experienced by marginalized communities, mixed‐methods implementation research is needed to engage families and codevelop context‐specific solutions, followed by cost‐effective scale‐up of effective policies and programmes.
- To improve awareness about breastfeeding among health practitioners and the public, and support evidence‐informed advocacy efforts, researchers should develop and adapt messaging for diverse stakeholders.
- To generate much needed knowledge about alternative methods of HM feeding (e.g., pumping, donor milk and milk sharing), researchers should accurately capture feeding practices.
- To evaluate the causal health effects of breastfeeding and HM, studies should be rigorously designed, carried out, analysed and interpreted to mitigate bias.
- To advance our knowledge of HM composition, synthesis and consumption, it is essential to apply standardized and validated sampling and analytical methods, to evaluate milk as a whole instead of a mixture of discrete components and to measure the volume of milk consumed accurately.
- To support evidence‐informed advocacy efforts, researchers should provide clear evidence summaries of their findings, discredit unsubstantiated claims and actively participate in initiatives leading to effective public policy and advocacy recommendations.

### 4.2 Call to action

Dissonance between groups in the breastfeeding and HM sector detracts from the energy and resources that advocates, researchers, health professionals and policymakers should be directing towards advancing a collective goal of supporting families and improving maternal–child health. Although it is understandable that members of the diverse breastfeeding advocacy and research communities will not always agree, they should endeavour to work together, not against each other, to advance this effort. To alleviate this conflict, *we call on individuals, companies and advocacy groups* to abstain from ad hominem attacks on HM and breastfeeding researchers and invest in developing a reasonable COI framework for effective governance of research in the field.

Further, *we call on governments and non‐profit organizations* to invest more in breastfeeding and HM research. We are encouraged that the US National Institutes of Health recently held a dedicated workshop on HM composition (Casavale et al., 2020) and launched a Task Force on Research Specific to Pregnant Women and Lactating Women. To support the development of updated Dietary Reference Intakes for infants, the US National Academies of Sciences, Engineering, and Medicine has created the Committee on Scanning for New Evidence on the Nutrient Content of Human Milk. We are also encouraged that philanthropic foundations have invested in implementation research to study policies and practices that support breastfeeding outcomes.

Finally, *we call on researchers* to embrace interdisciplinary initiatives to learn fresh perspectives, acquire new expertise and explore new applications for breastfeeding and HM research. Addressing outcomes beyond immediate infant health indicators (e.g., childhood educational performance, maternal health, environmental impacts and HM components as therapeutics for adult diseases) may encourage larger research initiatives focused on holistic and long‐term impacts of breastfeeding and HM. Research efforts are also needed to help mothers overcome lactational challenges, understand lactational failure and create an evidence base for donor milk provision in these cases.

## 5 CONCLUSION

**FIGURE 3:**
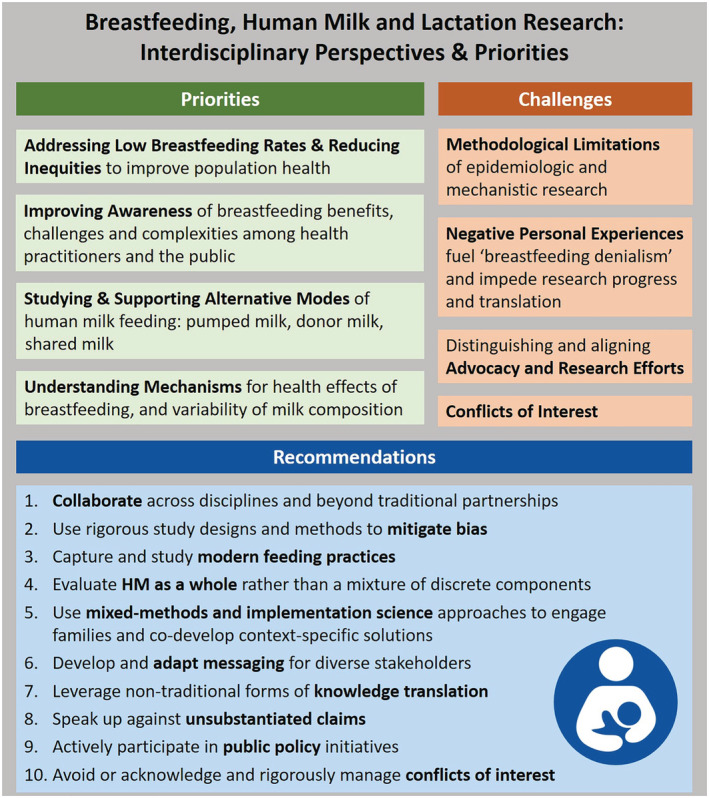
Key priorities and anticipated breakthroughs, barriers and challenges, and recommendations for research on breastfeeding and human milk (HM)

Breastfeeding and HM research is vital to understanding and improving health worldwide. As summarized in Figure 3, this transdisciplinary field is on the cusp of major discoveries with implications for lifelong health. However, unlike many other areas of health research, this field is laden with emotion and denialism. It is also challenged with informing yet remaining distinct, to some extent, from breastfeeding advocacy efforts. To advance research in this field and maximize its reach and impact, larger research investments are needed and interdisciplinary collaboration is essential; the scientific community must properly manage COI and engage families and other stakeholders in research planning and knowledge translation efforts.

## CONFLICTS OF INTEREST

The authors have declared all relevant potential sources of conflict of interest, including salary/research funding, patents, stock ownership, speaking activities at sponsored conferences/workshops/events, consulting, boards, societies, committees, expert panels and community organizations, detailed in Table S1.

## CONTRIBUTIONS

MBA and NCN planned and facilitated the workshop. All authors actively participated in workshop discussions, which formed the basis for this paper. The primary authors for Section 2.1 were ALP, RP‐E and NCN; Section 2.2: MB, AB, CG, KH and LZ; Section 2.3: BEY, CC, KMR and NS; Section 2.4: DM, MBA, LB and MM; Section 3.1: LZ, MBA, LB, DM and BEY; Section 3.2: NCN, MB and MBA; Section 3.3: DM, RP‐E, KH, LZ, MB and MM; and Section 3.4: NS, MM, RP‐E and NCN. MBA and NCN compiled and edited the individual sections with assistance from MB. All authors reviewed and revised the full manuscript and approved the final version for submission.

## Supporting information


**Table S1**. Potential conflicts of interest for all authors (last 24 months)
