# Supplementary material for: Breastfeeding and the origins of health: Interdisciplinary perspectives and priorities
Source: Matern Child Nutr. 2020 Nov 19;17(2):e13109. doi: 10.1111/mcn.13109 (PMC7988860; doi:10.1111/mcn.13109)
Supplement: Supplementary file 1 — Table S1. Potential conflicts of interest for all authors (last 24 months) [file MCN-17-e13109-s001.docx]

**Supplementary Figure 1**: Infants follow-up diagram


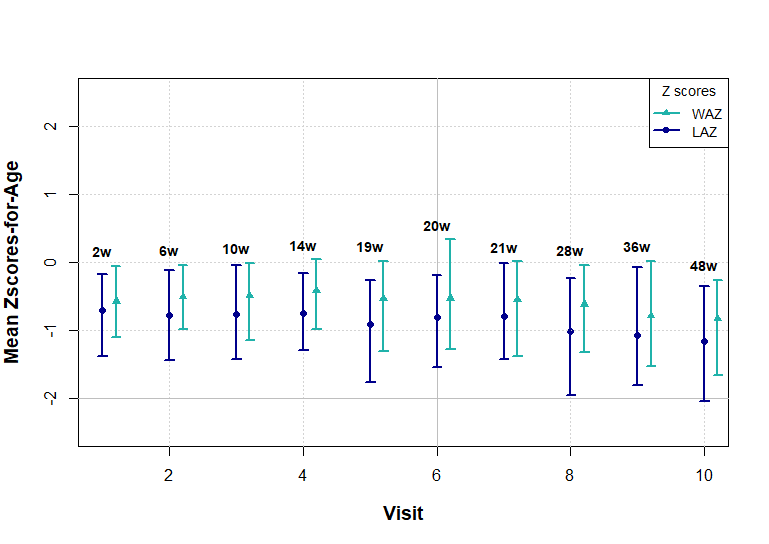


**Supplementary Figure 2:** Mean Weight-for-age and Length-for-age Z-scores for the whole cohort of infants eligible at birth with upper and lower quartiles.


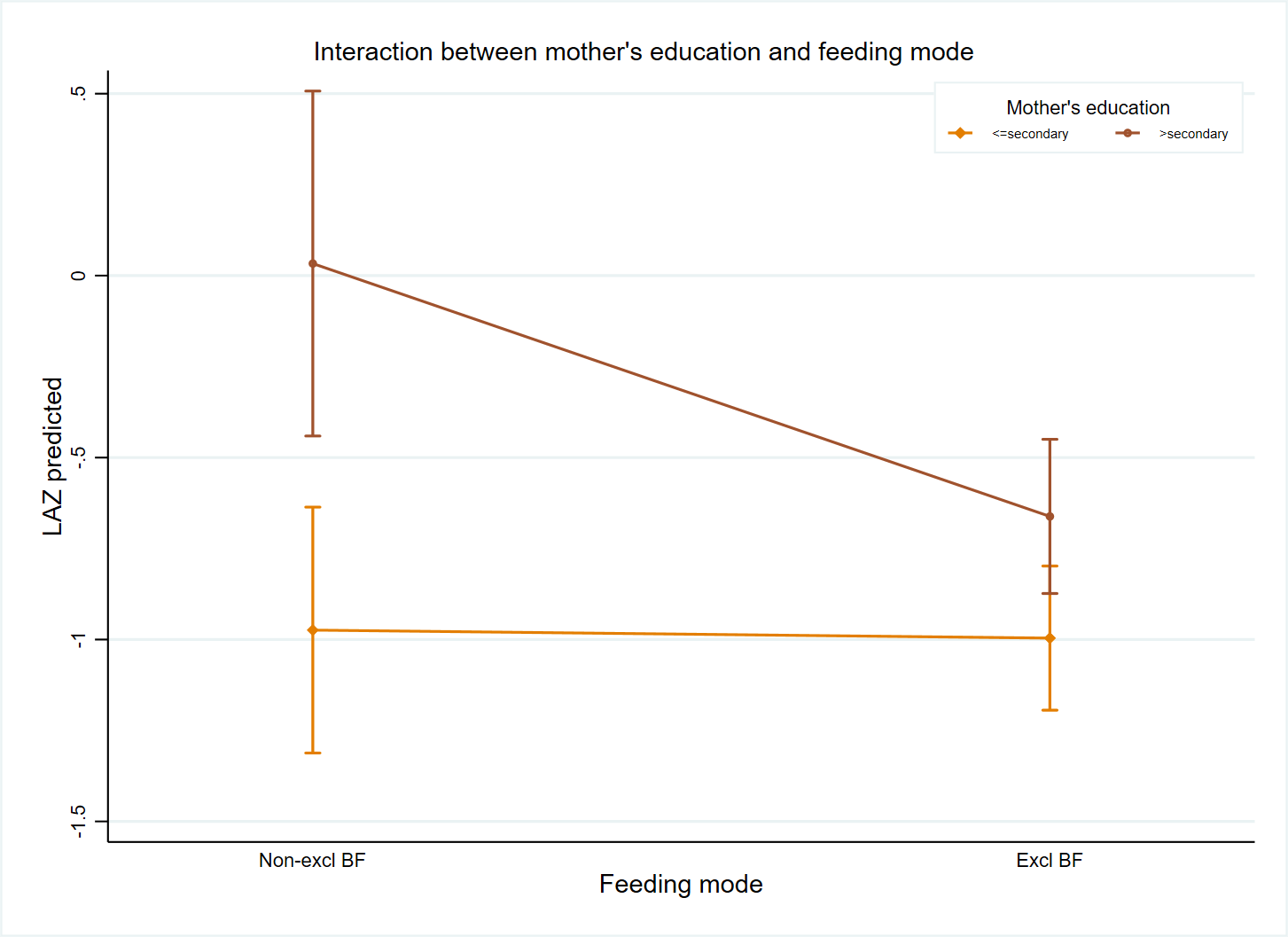


***Supplementary Figure 3****: Plot of interaction between mother's education and feeding mode in their effect on LAZ.*


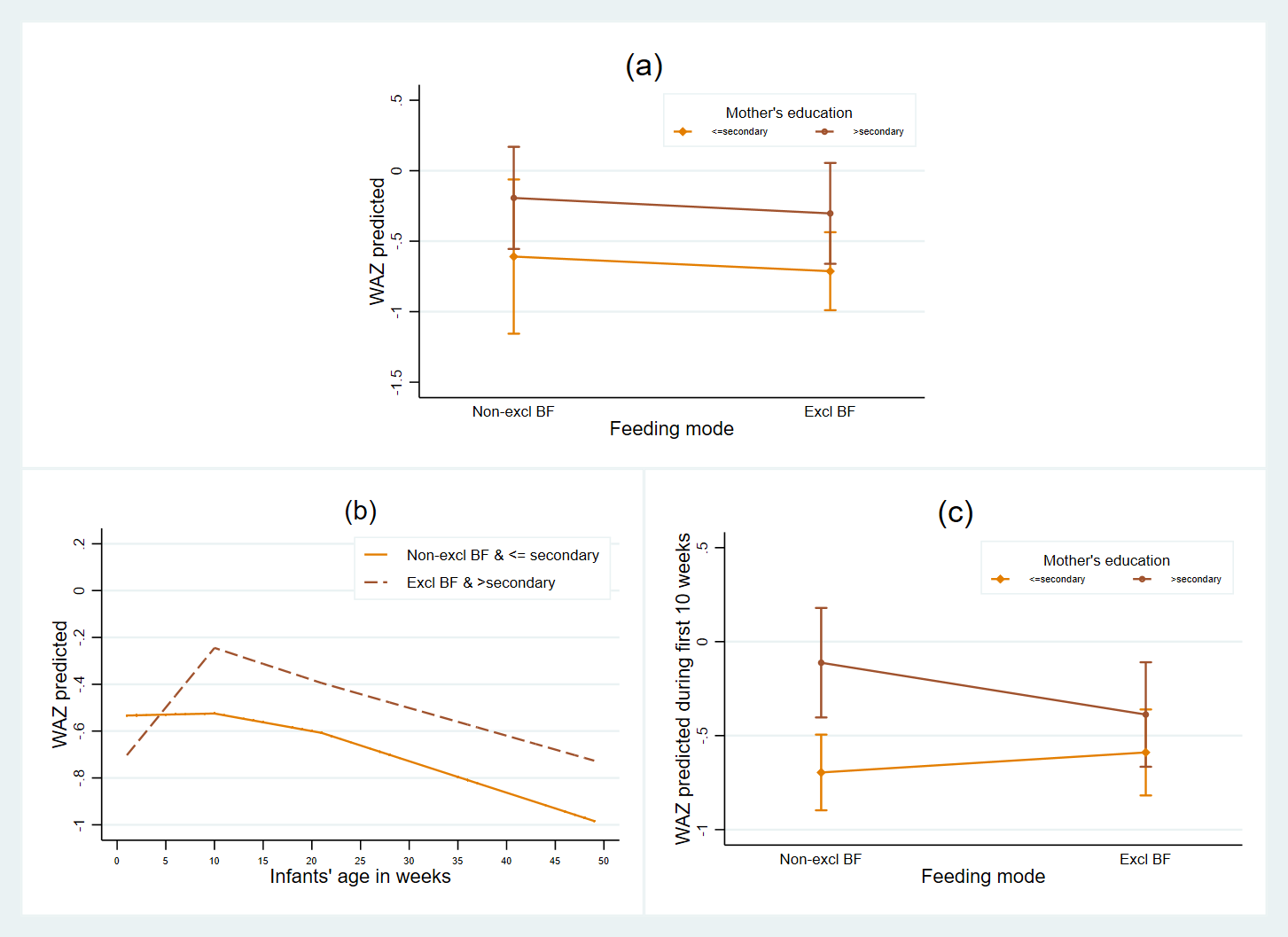


***Supplementary Figure 4****: Plots of interaction between mother's education and feeding mode:*

*(a) WAZ, (b) WAZ over time,* ***(c)*** *WAZ during first 10 weeks.*
